# Rare and population-specific functional variation across pig lines

**DOI:** 10.1186/s12711-022-00732-8

**Published:** 2022-06-03

**Authors:** Roger Ros-Freixedes, Bruno D. Valente, Ching-Yi Chen, William O. Herring, Gregor Gorjanc, John M. Hickey, Martin Johnsson

**Affiliations:** 1 The Roslin Institute and Royal (Dick) School of Veterinary Studies, The University of Edinburgh, Easter Bush, Midlothian, Scotland UK; 2 Departament de Ciència Animal, Universitat de Lleida - Agrotecnio-CERCA Center, Lleida, Spain; 3 The Pig Improvement Company, Genus plc, Hendersonville, TN USA; 4 Department of Animal Breeding and Genetics, Swedish University of Agricultural Sciences, Uppsala, Sweden

## Abstract

**Background:**

It is expected that functional, mainly missense and loss-of-function (LOF), and regulatory variants are responsible for most phenotypic differences between breeds and genetic lines of livestock species that have undergone diverse selection histories. However, there is still limited knowledge about the existing missense and LOF variation in commercial livestock populations, in particular regarding population-specific variation and how it can affect applications such as across-breed genomic prediction.

**Methods:**

We re-sequenced the whole genome of 7848 individuals from nine commercial pig lines (average sequencing coverage: 4.1×) and imputed whole-genome genotypes for 440,610 pedigree-related individuals. The called variants were categorized according to predicted functional annotation (from LOF to intergenic) and prevalence level (number of lines in which the variant segregated; from private to widespread). Variants in each category were examined in terms of their distribution along the genome, alternative allele frequency, per-site Wright’s fixation index (F_ST_), individual load, and association to production traits.

**Results:**

Of the 46 million called variants, 28% were private (called in only one line) and 21% were widespread (called in all nine lines). Genomic regions with a low recombination rate were enriched with private variants. Low-prevalence variants (called in one or a few lines only) were enriched for lower allele frequencies, lower F_ST_, and putatively functional and regulatory roles (including LOF and deleterious missense variants). On average, individuals carried fewer private deleterious missense alleles than expected compared to alleles with other predicted consequences. Only a small subset of the low-prevalence variants had intermediate allele frequencies and explained small fractions of phenotypic variance (up to 3.2%) of production traits. The significant low-prevalence variants had higher per-site F_ST_ than the non-significant ones. These associated low-prevalence variants were tagged by other more widespread variants in high linkage disequilibrium, including intergenic variants.

**Conclusions:**

Most low-prevalence variants have low minor allele frequencies and only a small subset of low-prevalence variants contributed detectable fractions of phenotypic variance of production traits. Accounting for low-prevalence variants is therefore unlikely to noticeably benefit across-breed analyses, such as the prediction of genomic breeding values in a population using reference populations of a different genetic background.

**Supplementary Information:**

The online version contains supplementary material available at 10.1186/s12711-022-00732-8.

## Background

Genetic variation is the basis of selective breeding in livestock and crop species. From a molecular point of view, genetic variants that result in either altered protein structures or altered gene expressions are believed to be responsible for much of the existing genetic variation for complex traits [1–4]. Missense variants change one amino acid of the encoded protein. Loss-of-function variants (LOF) are predicted to disrupt protein-coding transcripts such that they will not be translated into proteins or that they will be translated into non-functional proteins. Loss-of-function variants may change one amino acid codon into a premature stop codon (nonsense variants), change the reading frame during translation (frameshift indels), or change mRNA splicing (splicing variants). As such, potentially functional variants in protein-coding regions are assumed to be easier to detect (e.g., by association analyses) than variants that moderate gene expression [5–7]. Thus, missense and LOF variants are typically prioritised as putative causal variants for traits of interest (e.g., [8–11]).

Missense and LOF mutations can be pathogenic. For instance, missense and nonsense variants account for 57% of the entries in the Human Gene Mutation Database [12] (accessed on 30 April 2021), while small indels account for 22% and splicing variants account for another 9%. Similarly, in livestock species, many missense and LOF variants have been described as causal of genetic diseases and post-natal defects ([13–16]; Online Mendelian Inheritance in Animals [17], accessed on 30 April 2021), embryonic lethality [18, 19], or product defects [20, 21]. Deleterious missense and LOF variants are subject to purifying selection and are more likely to be rare, because they are related to unfavourable phenotypes such as disease risk or reduced fertility.

However, some missense and LOF mutations can be beneficial [22]. Moreover, some alleles that would be detrimental in the wild may be preferred in artificial selection settings. The artificial selection that is performed in livestock and crop breeding programs is expected to increase the frequency of alleles that favourably affect the traits included in the selection objectives. Therefore, it is also expected that missense and LOF variants are responsible for differences between breeds, genetic lines, and varieties of livestock and crop species that have undergone diverse selection histories. Identification of such functional variants can have direct applications in gene-assisted and genomic selection [23–25]. Furthermore, strategies for genetic improvement using genome editing have been theorized to either promote favourable alleles [26] or remove deleterious alleles [27] in selection candidates. Nevertheless, there is still limited knowledge about the presence of missense and LOF variants in commercial livestock populations, in particular regarding population-specific variants, often referred to as ‘private’, and how the presence of population-specific functional variants can affect applications such as across-breed genomic prediction.

Next-generation sequencing holds great potential for livestock breeding. One of its main benefits is the power to detect large numbers of variants, many of which will be specific to the population under study. A large number of individuals must, however, be sequenced in order to achieve high variant discovery rates, particularly for low-frequency variants [28, 29]. Several sequencing studies have profiled genomic variation in pigs [30–32], cattle [33, 34], or chicken [35]. These studies involved the sequencing of a small number of individuals (up to a few hundreds) at intermediate or high sequencing coverage. Alternatively, low sequencing coverage allows affordable sequencing of a much larger number of individuals, which would enable the identification of a much larger number of variants.

The objective of this study was to characterize the genetic variants in nine intensely selected pig lines with diverse genetic backgrounds. Particular emphasis was given to quantifying rare and population-specific functional variants, as well as the number of missense and LOF variants that an average individual carries. We also assessed the contribution of population-specific functional variants to the phenotypic variance of production traits.

## Methods

### Populations and sequencing strategy

We re-sequenced the whole genome of 7848 pigs from nine commercial lines (Genus PIC, Hendersonville, TN), with a total sequencing coverage of approximately 32,114×. Breeds of origin of the nine lines were Large White, Landrace, Pietrain, Hampshire, Duroc, and synthetic lines. The number of pigs that were available in the pedigree of each line and the number of sequenced pigs, by coverage, are summarized in Table 1.

**Table 1:** Number of sequenced and analysed pigs

Line	Individuals sequenced	Individuals sequenced by coverage				Individuals used in analyses		
		1×	2×	5×	15–30×	Pedigree	Imputed	GWAS
A	1856	1044	649	73	90	122,753	104,661	88,342
B	1491	628	728	54	81	84,420	66,608	56,173
C	1366	685	545	44	92	88,964	76,230	64,285
D	760	394	274	27	65	50,797	41,573	–
E	731	362	311	16	42	79,981	60,474	–
F	701	351	255	28	67	52,470	39,263	–
G	445	217	176	15	37	21,129	17,224	–
H	381	193	137	16	35	35,309	29,330	–
I	321	111	158	18	34	15,495	5247	–

Which pigs to sequence and their coverage were determined following a three-part sequencing strategy, with the objective of representing the haplotype diversity in each line. First (1), top sires and dams with the largest number of genotyped progeny were sequenced at 2× and 1×, respectively. Sires were sequenced at higher coverage because they individually contributed more progeny than dams. Then (2), individuals with the greatest genetic footprint on the population (i.e., those that carry more of the most common haplotypes) and their immediate ancestors were sequenced at a target sequencing coverage between 1× and 30×, as assigned by an algorithm that maximises the expected phasing accuracy of the common haplotypes from the accumulated family information (AlphaSeqOpt part 1; [36]). Finally (3), pigs that carried haplotypes with a low accumulated coverage (below 10×) were sequenced at 1× (AlphaSeqOpt part 2; [37]). Sets (2) and (3) were based on haplotypes inferred from marker array genotypes (GGP-Porcine HD BeadChip; GeneSeek, Lincoln, NE), which were phased using AlphaPhase [38] and imputed using AlphaImpute [39]. As a result of this sequencing strategy, sequencing effort in each of the nine lines was proportional to their population size, at approximately 1.5% (0.9–2.1%) of the pigs in each line. Most pigs were sequenced at a low target coverage of 1 or 2×. The average individual coverage was 4.1×, but the median coverage was 1.5×. Population structure across the nine lines was assessed with a principal component analysis using the sequenced pigs and is shown in Additional file 1: Fig. S1.

Most sequenced pigs, as well as pedigree relatives, were also genotyped with marker arrays either at low density (15k markers) using the GGP-Porcine LD BeadChip (GeneSeek) or at high density (50k or 80k markers) using different versions of the GGP-Porcine HD BeadChip (GeneSeek).

### Sequencing and data processing

Tissue samples were collected from ear punches or tail clippings. Genomic DNA was extracted using Qiagen DNeasy 96 Blood & Tissue kits (Qiagen Ltd., Mississauga, ON, Canada). Paired-end library preparation was conducted using the TruSeq DNA PCR-free protocol (Illumina, San Diego, CA). Libraries for resequencing at low coverage (1 to 5×) were produced with an average insert size of 350 bp and sequenced on a HiSeq 4000 instrument (Illumina). Libraries for resequencing at high coverage (15 or 30×) were produced with an average insert size of 550 bp and sequenced on a HiSeq X instrument (Illumina). All libraries were sequenced at Edinburgh Genomics (Edinburgh Genomics, University of Edinburgh, Edinburgh, UK).

DNA sequence reads were pre-processed using the Trimmomatic software [40] to remove adapter sequences and then aligned to the reference genome *Sscrofa11.1* (GenBank accession: GCA_000003025.6) using the BWA-MEM algorithm [41]. Duplicates were marked using the Picard software (http://broadinstitute.github.io/picard). Single nucleotide polymorphisms (SNPs) and short insertions and deletions (indels) were identified with GATK HaplotypeCaller (GATK 3.8.0) [42, 43] using default settings. Variant discovery was performed separately for each individual and then a joint variant set for each population was obtained by extracting the variant positions from all sequenced individuals. Between 20 and 30 million variants were discovered in each population.

Read counts supporting each allele were directly extracted from the aligned reads stored in the BAM files using a pile-up function in order to avoid biases towards the reference allele that are introduced by the GATK algorithm when applied on low-coverage whole-genome sequence data [44]. This pipeline uses pysam (version 0.13.0; https://github.com/pysam-developers/pysam), which is a wrapper around htslib and the samtools package [45]. We extracted the read counts for all biallelic variant positions, after filtering variants in potential repetitive regions with the VCFtools software [46]. Variants in potential repetitive regions were defined as those that had a mean depth value that was 3 times greater than the average realized coverage. In total, 46,344,624 biallelic variants passed quality control criteria in at least one of the nine lines (see Additional file 2: Supplementary Methods).

### Genotype imputation

Genotypes were jointly called, phased and imputed for a total of 537,257 pedigree-related individuals across lines, using the ‘hybrid peeling’ method implemented in AlphaPeel [47–49], which used all available SNP panels and whole-genome sequence data. Imputation was performed separately for each line using its complete multi-generational pedigree, which encompassed from 15,495 to 122,753 individuals each (Table 1). We have previously published on the accuracy of imputation in the same populations using this method [48]. The estimated average allele dosage correlation (correlation between the real genotype and the imputed allele dosage) by individual was 0.94 (median 0.97) [48]. Individuals with a low predicted imputation accuracy were removed before further analyses. An individual was predicted to have a low imputation accuracy if itself or all its grandparents were not genotyped with a marker array or if it had a low degree of connectedness to the rest of the population (defined as the sum of coefficients of relationship between the individual and the rest of individuals in the pedigree). These criteria were based on analysis of simulated and real data on imputation accuracy [48]. In total, 440,610 individuals remained, from 5247 to 104,661 individuals for each line (Table 1). The expected average individual-wise dosage correlation of the remaining individuals was 0.97 (median 0.98) [48]. Although variants with a minor allele frequency lower than 0.023 had an estimated variant-wise dosage correlation lower than 0.90 [48], in our analyses, we did not filter variants based on minor allele frequency to account for the whole frequency spectrum.

### Variant predicted consequence types

The frequency of the alternative allele was calculated based on the imputed genotypes. The prevalence level of a variant was defined as the number of lines in which the variant segregated. To differentiate allele frequency and prevalence level, we used the terms ‘rare’ and ‘common’ to refer to variants in terms of allele frequency and ‘private’ and ‘widespread’ in terms of prevalence level, where private variants were those called only in one line and widespread variants those called in all nine studied lines. We calculated Wright’s fixation statistic (F_ST_) [50] for each variant among the lines in which the variant segregated as F_ST_ = (H_T_ − H_S_)/H_T_, where H_T_ is the expected heterozygosity across the lines under Hardy–Weinberg equilibrium and H_S_ is the average heterozygosity within lines under Hardy–Weinberg equilibrium.

Variants were annotated using Ensembl Variant Effect Predictor (Ensembl VEP; version 97, July 2019) [51] using both Ensembl and RefSeq transcript databases. For variants with multiple predicted consequence types (e.g., in the case of multiple transcripts), the variant was annotated with the most severe predicted consequence type. Stop-gain, start-loss, stop-loss, splice donor, and splice acceptor variants were classified as LOF variants. Although frameshift indels are typically included in the LOF category, we considered them as a separate category in order to quantify their impact separately. The SIFT scores [52] for missense variants were retrieved from the Ensembl transcript database. Missense variants for which SIFT scores were available were then classified as deleterious when their SIFT score was less than 0.05 and as tolerated otherwise. We considered the predicted consequence types of LOF, frameshift and in-frame indels, and missense variants as putatively functional. To account for the regulatory role of promoters, we classified variants within 500 bp upstream of the annotated transcription start site in the same consequence type as the variants in the 5′ untranslated region (UTR) because both these regions likely contain regulatory elements that affect transcription and because the same variant can be in the promoter and in the 5′ UTR of different annotated transcripts for the same gene. As a result, 6.6% of the variants that were initially classified by Ensembl VEP as ‘variants upstream of gene’, were reclassified as ‘variants in promoter regions’. For further analyses, variants in promoters and in the 5′ and 3′ UTR were jointly considered (Promoter + UTR). Because some variants, such as stop-gain (LOF) variants or frameshift indels, are more likely to be benign when located towards the end of the transcripts (e.g., [53]), we also analysed the relative position of these variants within transcripts (i.e., position accounting for transcript length).

### Load of putatively functional alleles

We used the imputed genotypes to estimate the average number of alleles of each predicted consequence type and prevalence level that an individual carried. For the most common predicted consequence types, this was estimated from 50,000 randomly sampled variants. For tolerated missense variants, we used the 50,000 variants with the highest SIFT scores. To account for the different number of variants for each predicted consequence type and prevalence level category, we calculated the heterozygosity and homozygosity for the alternative allele for each individual as the percentage of variants of each category that the individual carried, respectively, in the heterozygous state and the homozygous state for the alternative allele.

### Association to production traits

To further explore the association of variants in each predicted consequence type and prevalence level category with production traits, we performed genome-wide association studies (GWAS) for the three largest lines, using all the called variants that passed filtering. We chose average daily gain, backfat thickness, and loin depth because they are complex traits with moderate heritability estimates (from 0.21 to 0.38). The number of pigs with records that were included in the GWAS are in Table 1. Most pigs with records were born during the 2008–2020 period. Breeding values were estimated by line with a linear mixed model that included polygenic effects and the non-genetic effects of contemporary group, litter, and body weight, as relevant for each trait. Deregressed estimated breeding values were obtained following the method of VanRaden et al. [54]. Only individuals for which the trait was directly measured were retained for the GWAS, by fitting the following univariate linear mixed model using the FastLMM software [55, 56]:

$$\mathbf{y}= {\mathbf{x}}_{i}{\upbeta }_{i}+\mathbf{u}+\mathbf{e},$$

where $\mathbf{y}$ is the vector of deregressed estimated breeding values, ${\mathbf{x}}_{i}$ is the vector of genotypes for the $i$th variant, coded as 0 and 2 if homozygous for either allele or 1 if heterozygous, ${\upbeta }_{i}$ is the allele substitution effect of the $i$th variant on the trait, $\mathbf{u}\sim N(0,{\upsigma }_{\mathrm{u}}^{2}\mathbf{K})$ is the vector of polygenic effects with the covariance matrix equal to the product of the polygenic additive genetic variance ${\upsigma }_{\mathrm{u}}^{2}$ and the genomic relationship matrix $\mathbf{K}$, and $\mathbf{e}$ is a vector of uncorrelated residuals. Due to computational limitations, the genomic relationship matrix $\mathbf{K}$ was calculated using imputed genotypes for the high-density marker array and its single-value decomposition was taken.

We considered associations with a p-value equal or smaller than 10^–6^ as significant. We calculated an enrichment score for each predicted consequence type and prevalence level category as:

$$\mathrm{log}\left(\frac{\mathrm{nSignCategory}/\mathrm{nNotSignCategory}}{\mathrm{nSignTotal}/\mathrm{nNotSignTotal}}\right),$$

where $\mathrm{nSignCategory}$ is the number of variants with a significant association with at least one trait in one of the three lines for a given predicted consequence type and prevalence level category, $\mathrm{nNotSignCategory}$ is the number of variants with no significant association in the same category, and $\mathrm{nSignTotal}$ and $\mathrm{nNotSignTotal}$ are the total numbers of variants with and without significant association, respectively.

Linkage disequilibrium is pervasive between nearby significant variants due to the extremely high variant density of whole-genome sequence data. To account for this, we defined haplotype blocks and considered only one variant per haplotype block as the putative driver of an association that was detected in that region. We defined the haplotype blocks for each line separately using the—*blocks* function in Plink 1.9 [57, 58] by considering pairs of variants within 5 Mb of each other to be in strong linkage disequilibrium if the bottom of the 90% confidence interval of D’ was greater than 0.7 and the top of the confidence interval was at least 0.9. If the top of the confidence interval was smaller than 0.7, it was considered as strong evidence for historical recombination between the two variants. All other pairs of variants were considered uninformative. Regions for which at least 90% of the informative pairs of variants showed strong linkage disequilibrium were defined as a haplotype block. Within each haplotype block, we selected the variant with the most severe predicted consequence type as the candidate variant, as a simplification of common assumptions in the prioritisation of candidate variants. If there was more than one variant with the same predicted consequence type, the one with the lowest p-value was selected. This process was performed separately for each trait and line.

We calculated the additive genetic variance explained by each variant as 2*pq*${\widehat{\beta }}^{2}$, where *p* and *q* were the allele frequencies and $\widehat{\beta }$ is the estimated allele substitution effect of the variant. We expressed the variance explained by each variant as a percentage of the phenotypic variance of each trait. Finally, we calculated the median F_ST_ of the candidate variants within each predicted consequence type and prevalence level category and compared it to the median F_ST_ of the same category as the logarithm of the ratio of the former to the latter.

## Results

### Prevalence of variants

A large percentage (21%) of the 46,344,624 biallelic variants that passed quality control criteria were widespread in all nine lines. Private variants represented a much smaller percentage (2 to 11%) of the variants called within each line. However, when counted across lines, private variants cumulatively predominated (28%) over the widespread ones. Most variants were neither private nor widespread. The distribution of these variants by line is shown in Table 2. Most variants (38,642,777) were SNPs, of which 10,595,681 were called in a single line (27%; 366,486 to 2,743,965 within each line) and 8,377,578 (22%) were called in all nine lines. The remaining 7,701,847 variants were indels, of which 2,436,674 were called in a single line (32%; 121,525 to 506,149 in each line) and 1,560,353 (20%) were called in all nine lines.

**Table 2:** Number of variants by line

Line	Biallelic variant sites (× 10^6^)	SNPs			Indels		
		All biallelic (× 10^6^)	Private (× 10^6^)	Widespread (× 10^6^)	All biallelic (× 10^6^)	Private (× 10^6^)	Widespread (× 10^6^)
A	28.83	24.38	1.56	8.38	4.44	0.39	1.56
B	28.57	24.32	2.74	8.38	4.24	0.51	1.56
C	28.88	24.60	2.51	8.38	4.28	0.44	1.56
D	21.44	17.94	1.23	8.38	3.50	0.32	1.56
E	19.06	15.71	0.51	8.38	3.35	0.22	1.56
F	20.21	16.86	0.42	8.38	3.35	0.16	1.56
G	23.38	19.64	0.50	8.38	3.74	0.16	1.56
H	22.32	18.78	0.37	8.38	3.55	0.12	1.56
I	24.59	20.82	0.76	8.38	3.77	0.13	1.56
Total	46.30	38.64	10.60	8.38	7.70	2.44	1.56

### Distribution of variants and relationship with recombination rate

**Fig. 1:**
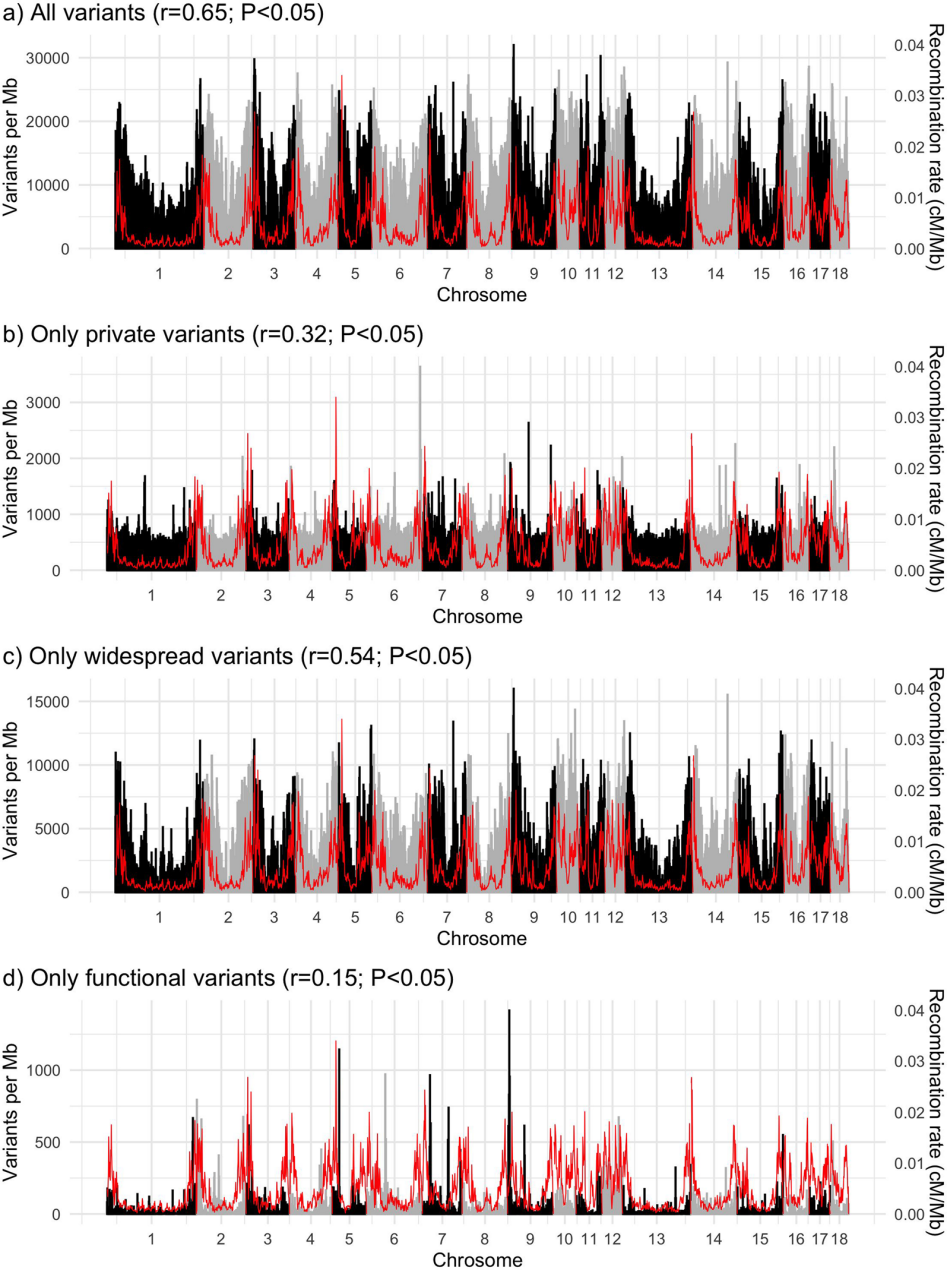
Relationship of variant density in line A (black and grey bars) with recombination rate (red line). The correlation (r) between variant density and recombination rate in 1-Mb non-overlapping windows is reported

The number of variants by chromosome was strongly correlated with chromosome length (r = 0.98, P < 0.05) (see Additional file 3: Table S1). The variant density by chromosome was negatively correlated with chromosome length (r = − 0.87, P < 0.05) and (see Additional file 3: Table S1). The variant density within 1-Mb non-overlapping windows was positively correlated with recombination rate in that window (r = 0.65, P < 0.05; Fig. 1a) [59]. For example, in line A, there was on average one variant every 81 bp, but in the 5% 1-Mb windows with the lowest and highest recombination rates there was on average one variant every 152 and 54 bp, respectively (2.8-fold more variants in windows with high recombination rate than in windows with low recombination rate). Across all lines, there was one variant every 49 bp on average, but in the 5% 1-Mb windows with the lowest and highest recombination rates there was on average one variant every 79 and 34 bp, respectively (2.3-fold more variants in windows with high recombination rate).

The distribution of private and widespread variants along the genome also differed. The density of widespread variants was more correlated with recombination rate than the density of private variants (Fig. 1b and c). As a consequence, private variants represented a larger proportion of the variation in regions with low recombination rate, which were depleted of widespread variants. Across all lines, in the 5% 1-Mb windows with the highest recombination rates there was on average one private variant every 167 bp and one widespread variant every 148 bp (1.1-fold more private variants relative to widespread). In the 5% 1-Mb windows with the lowest recombination rates there was on average one private variant every 260 bp and one widespread variant every 531 bp (2.0-fold more private variants relative to widespread). There were no genomic regions that were enriched for private variants across the nine lines (see Additional file 4: Fig. S2).

### Frequency and fixation index

**Fig. 2:**
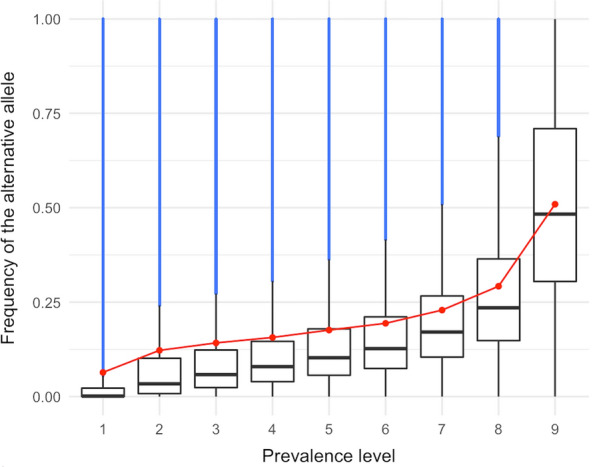
Frequency of the alternative allele by prevalence level. Red dots indicate means. In blue, values greater than 1.5 times the interquartile range

**Fig. 3:**
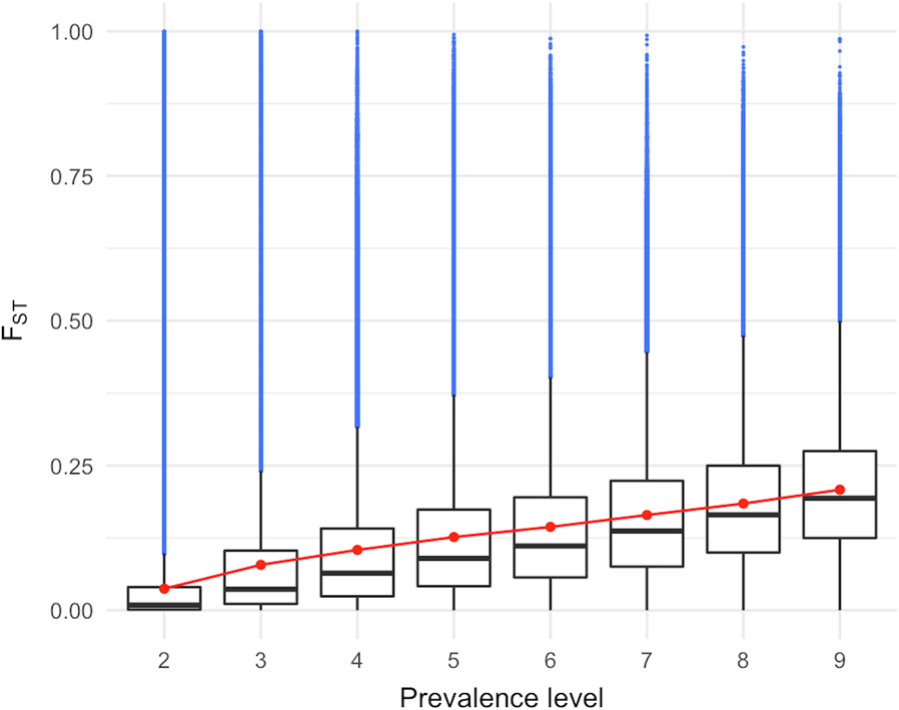
Wright’s fixation statistic (F_ST_) by prevalence level. Red dots indicate means. In blue, values greater than 1.5 times the interquartile range

The prevalence level and alternative allele frequency were related, in a way that less prevalent variants had a lower allele frequency (Fig. 2) and a lower F_ST_ (Fig. 3). Private variants had an average alternative allele frequency of 0.03 (SD = 0.09), as opposed to widespread variants, which had an average alternative allele frequency of 0.50 (SD = 0.25). Because the less prevalent variants generally had low alternative allele frequencies, they showed a small degree of differentiation between the lines in which they segregated (F_ST_ = 0.04, SD = 0.07). In contrast, widespread variants had the largest degree of differentiation between lines (F_ST_ = 0.21, SD = 0.11).

### Prevalence and frequency of putatively functional variants

The predicted consequence types of the variants are listed in Table 3. Half (49.9%) of the variants were called in intergenic regions and another 47.0% of the variants were called in intronic regions. Only 2.2% of the variants were called in the promoter or 5′ and 3′ UTR. The coding variants comprised 0.9% of the total variants, of which more than half were missense (45.5%), frameshift indels (3.1%) or LOF (3.7%). The density of putatively functional variants was only weakly correlated with recombination rate in 1-Mb non-overlapping windows (Fig. 1d).

**Table 3:** Predicted consequence types of variants by prevalence level

Consequence type	Percentage of variants (%) by prevalence level										r
	1	2	3	4	5	6	7	8	9	Total	
**Loss-of-function**^**a**^	**0.061**	**0.035**	**0.026**	**0.021**	**0.019**	**0.017**	**0.019**	**0.018**	**0.019**	**0.032**	**− .76**^**b**^
Splice acceptor/donor	0.038	0.023	0.014	0.010	0.009	0.007	0.008	0.008	0.008	0.018	**− **.79^b^
Stop-gain	0.014	0.009	0.008	0.008	0.007	0.007	0.007	0.006	0.006	0.009	**− .**82^b^
Stop-loss	0.005	0.002	0.002	0.002	0.002	0.002	0.002	0.002	0.003	0.003	**− **.36
Start-loss	0.004	0.002	0.002	0.002	0.001	0.001	0.002	0.002	0.002	0.002	**− **.47
**Frameshift indel**	**0.014**	**0.017**	**0.019**	**0.021**	**0.020**	**0.021**	**0.024**	**0.032**	**0.055**	**0.027**	**+ .81**^b^
**In-frame indel**	**0.005**	**0.008**	**0.009**	**0.008**	**0.008**	**0.008**	**0.007**	**0.007**	**0.005**	**0.006**	**− .23**
Missense	0.556	0.378	0.355	0.340	0.344	0.336	0.319	0.306	0.325	0.393	**− **.73^b^
**Deleterious**	**0.201**	**0.092**	**0.074**	**0.069**	**0.064**	**0.062**	**0.054**	**0.048**	**0.040**	**0.096**	**− .78**^b^
**Tolerated**	**0.223**	**0.170**	**0.165**	**0.165**	**0.173**	**0.167**	**0.161**	**0.159**	**0.177**	**0.183**	**− .52**
Splice region	0.105	0.098	0.088	0.081	0.083	0.081	0.080	0.081	0.085	0.090	**− **.76^b^
**Synonymous**	**0.240**	**0.313**	**0.334**	**0.348**	**0.355**	**0.353**	**0.337**	**0.331**	**0.353**	**0.316**	** + .65**
**Untranslated regions**	**2.300**	**2.252**	**2.257**	**2.191**	**2.146**	**2.156**	**2.093**	**2.089**	**2.061**	**2.180**	**− .98**^b^
Promoter + 5’ UTR	0.879	0.825	0.812	0.812	0.787	0.813	0.759	0.766	0.759	0.810	**− **.90^b^
3’ UTR	1.421	1.427	1.445	1.378	1.359	1.343	1.334	1.322	1.302	1.370	**− **.94^b^
Non-coding transcript exon	0.104	0.113	0.107	0.113	0.128	0.118	0.105	0.109	0.117	0.111	+ .25
**Intronic**	**47.744**	**47.571**	**47.634**	**47.162**	**46.513**	**46.709**	**46.701**	**46.355**	**46.132**	**46.981**	**− .95**^b^
Upstream of gene	3.062	3.066	3.075	3.041	3.083	3.056	2.929	2.943	2.936	3.015	**− **.81^b^
Downstream of gene	2.660	2.679	2.740	2.747	2.746	2.705	2.700	2.707	2.676	2.692	+ .04
**Intergenic**	**43.148**	**43.468**	**43.355**	**43.927**	**44.553**	**44.439**	**44.687**	**45.021**	**45.235**	**44.154**	**+ .97**^b^

The most severe consequence of each variant was used. The main Sequence Ontology (SO) terms are shown in order of severity (more severe to less severe) as estimated by Ensembl Variant Effect Predictor. The correlation (r) between the percentage of variants of each consequence type and prevalence is reported

In bold, categories that will be analysed in the next sections

^a^If frameshift indels were included in this category: r = −.06 (P > 0.05)

^b^Significant correlation (P < 0.05)

The low-prevalence variants (i.e., the variants that were identified in one or a few lines) were enriched for missense and LOF variants, as well as for potentially regulatory variants such as those located in the promoter and 5′ and 3′ UTR and other intronic variants. In contrast, the high-prevalence variants (i.e., the variants that were identified in many or all the lines) were enriched for frameshift indels and for synonymous (non-significant correlation) and intergenic variants. Although frameshift indels are typically included in the LOF category, our results show that the LOF category is very heterogeneous and the frameshift indels presented opposite patterns to other LOF variants. Therefore, we studied frameshift indels as a separate category.

**Fig. 4:**
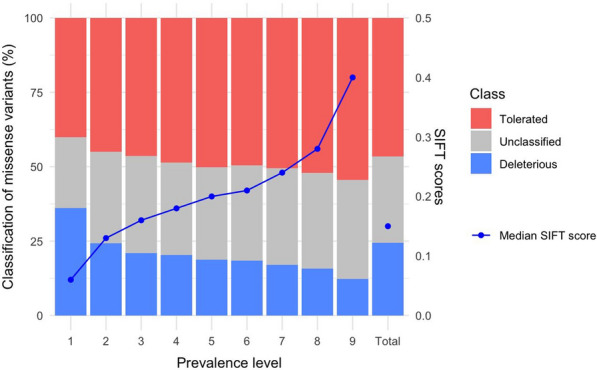
Classification of the missense variants and median SIFT score by prevalence level

**Fig. 5:**
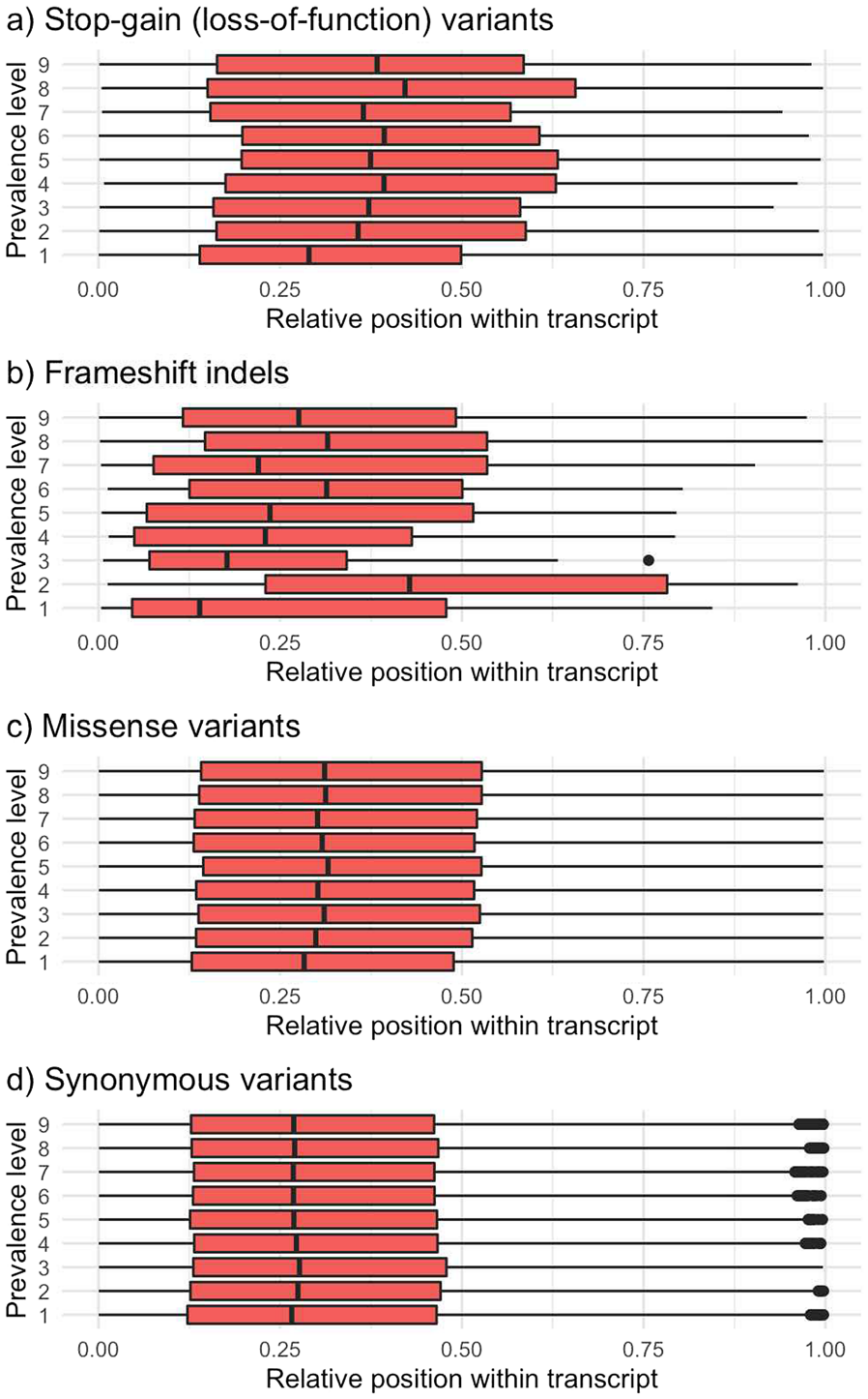
Relative position within transcript of stop-gain, frameshift indels, missense, and synonymous variants, by prevalence level

Although the LOF variants had lower allele frequencies than the intergenic variants when they had low prevalence, they had similar allele frequencies in high-prevalence levels (Table 4). Thus, there was a set of LOF variants that were prevalent across lines and that also had particularly high frequencies within lines. Missense variants had lower allele frequencies than the intergenic variants for all prevalence levels, especially those classified as deleterious. The low-prevalence missense variants were enriched for a larger fraction of deleterious variants and lower SIFT scores than high-prevalence missense variants (Fig. 4). Unlike missense or synonymous variants, low-prevalence stop-gain (LOF) variants and frameshift indels were more likely located towards the start of the transcripts (Fig. 5). In contrast to LOF and missense variants, frameshift and in-frame indels had intermediate allele frequencies, much higher than those of intergenic variants (Table 4), which indicated that the minor allele was the reference allele, in many cases. Within prevalence level, the LOF and deleterious missense variants had lower F_ST_ than the intergenic variants (Table 5), probably because they were kept at low allele frequencies due to negative selection pressure. Frameshift and in-frame indels also had lower F_ST_ than intergenic variants, in spite of their intermediate allele frequencies.

**Table 4:** Frequency of the alternative allele by predicted consequence type and prevalence level

Consequence type	Frequency of the alternative allele by prevalence level									
	1	2	3	4	5	6	7	8	9	Total
Loss-of-function	0.0010	0.017	0.048	0.062	0.089	0.114	0.151	0.223	0.489	0.020
Frameshift indel	0.4816	0.758	0.757	0.420	0.302	0.260	0.339	0.456	0.693	0.634
In-frame indel	0.8893	0.903	0.910	0.898	0.812	0.785	0.702	0.595	0.572	0.735
Deleterious missense	0.0006	0.018	0.043	0.061	0.078	0.092	0.125	0.170	0.350	0.010
Tolerated missense	0.0011	0.027	0.047	0.066	0.083	0.106	0.143	0.202	0.443	0.074
Synonymous	0.0037	0.032	0.049	0.066	0.086	0.107	0.151	0.205	0.447	0.110
Promoter + UTR	0.0019	0.034	0.059	0.078	0.099	0.122	0.166	0.226	0.475	0.102
Intronic	0.0015	0.035	0.059	0.080	0.102	0.126	0.171	0.235	0.485	0.110
Intergenic	0.0015	0.033	0.058	0.080	0.105	0.129	0.173	0.237	0.483	0.116

Values are medians

**Table 5:** Wright’s fixation statistic (F_ST_) by predicted consequence type and prevalence level

Consequence type	F_ST_ by prevalence level								
	2	3	4	5	6	7	8	9	Total
Loss-of-function	0.003	0.022	0.047	0.066	0.094	0.114	0.145	0.171	0.071
Frameshift indel	0.010	0.042	0.065	0.081	0.088	0.120	0.146	0.148	0.114
In-frame indel	0.011	0.035	0.051	0.070	0.087	0.105	0.115	0.130	0.077
Deleterious missense	0.005	0.029	0.055	0.073	0.087	0.110	0.131	0.160	0.068
Tolerated missense	0.009	0.036	0.061	0.084	0.107	0.127	0.158	0.184	0.108
Synonymous	0.013	0.040	0.062	0.090	0.110	0.130	0.158	0.194	0.117
Promoter + UTR	0.009	0.036	0.060	0.086	0.108	0.131	0.158	0.190	0.110
Intronic	0.009	0.037	0.063	0.089	0.111	0.136	0.164	0.195	0.118
Intergenic	0.009	0.036	0.066	0.091	0.112	0.139	0.167	0.193	0.121

Values are medians

### Load of putatively functional alleles by prevalence level

Most missense deleterious and LOF variants that an individual carried in the homozygous state for the alternative allele were high-prevalence variants. Only a small proportion of these variants were private. An individual carried on average 1048 (SD = 57) LOF variants in the homozygous state for the alternative allele, of which 713 (SD 36) were widespread across all nine lines and only 20 (SD = 7) were private. An average individual carried 1379 (SD = 165) deleterious missense variants in the homozygous state for the alternative allele, of which 1012 (SD = 79) were widespread and only 4 (SD = 3) were private. An average individual carried 1080 (SD = 89) LOF and 2632 (SD = 235) deleterious missense variants in the heterozygous state.

**Fig. 6:**
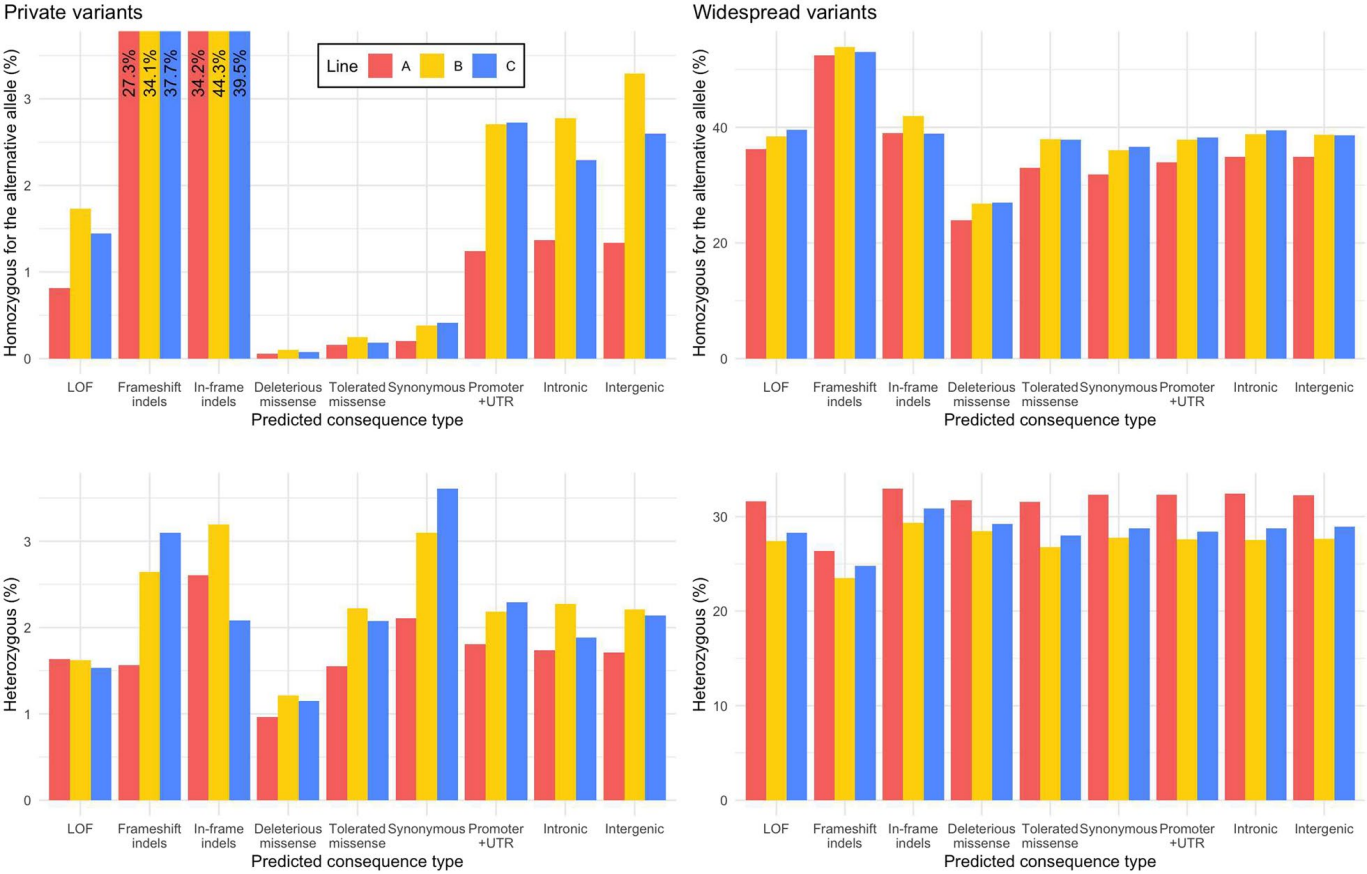
Average percentage of variants in the homozygous state for the alternative allele or in the heterozygous state across individuals by predicted consequence type and prevalence level. LOF: loss-of-function; UTR: untranslated regions

We found signals of negative selection against deleterious missense variants, in particular private ones. Individuals proportionally carried fewer deleterious missense variants in the homozygous state for the alternative allele than variants of other predicted consequence types, regardless of prevalence level (Fig. 6). Individuals also carried proportionally less private tolerated missense, synonymous and LOF variants in the homozygous state for the alternative allele than expected.

### Associations of low-prevalence variants with production traits

**Fig. 7:**
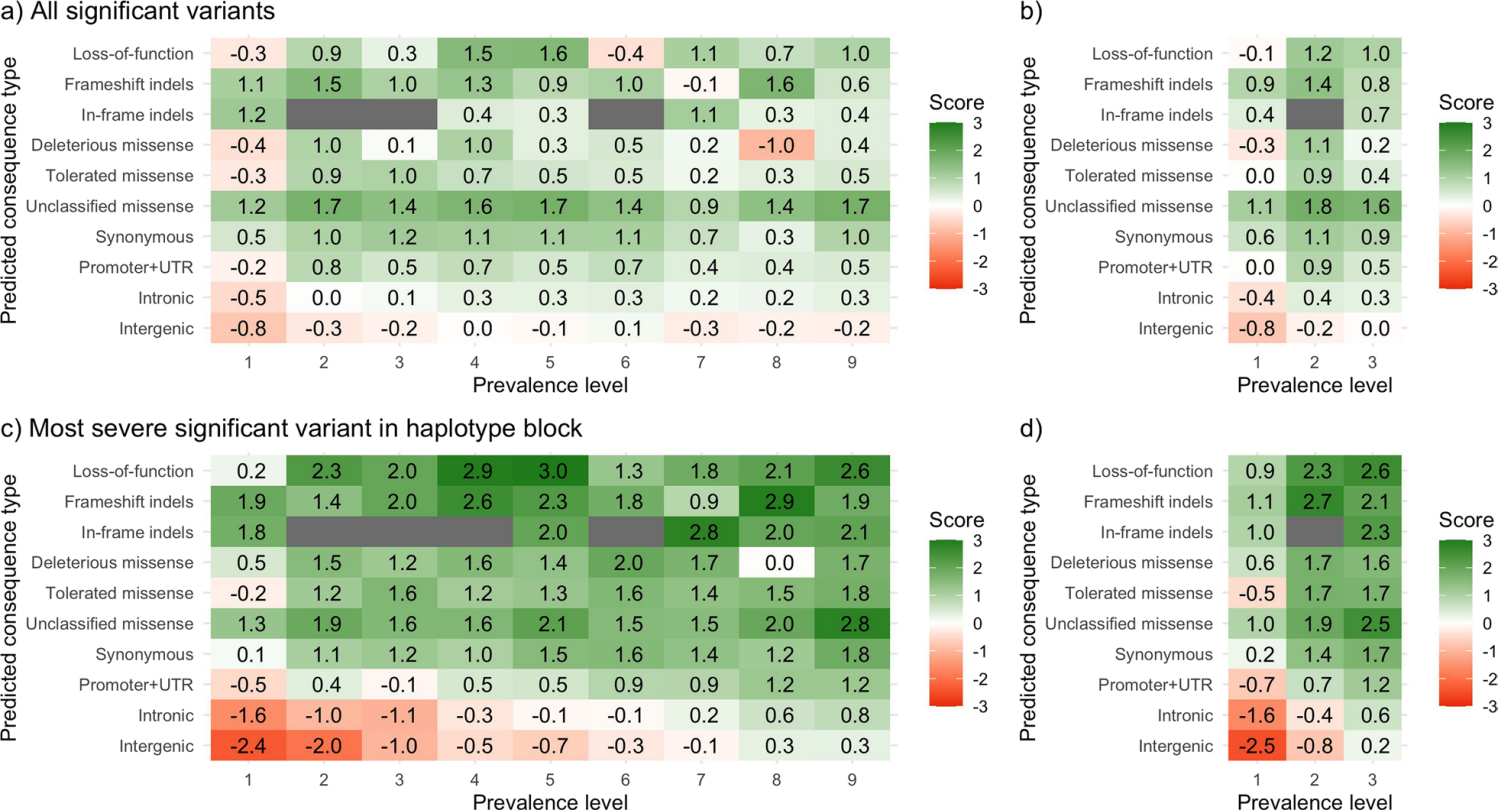
Enrichment score for the number of significant variants in the genome-wide association study by variant prevalence level and predicted consequence type. Either all significant variants (**a**, **b**) or only the most severe significant variants within haplotype blocks (**c**, **d**) were used. Prevalence level was considered across all nine lines (**a**, **c**) or only across the three lines included in the genome-wide association study (**b**, **d**)

Significant variants were enriched for putatively functional and regulatory variants of different prevalence levels, and depleted of intergenic variants. In total, 108,109 variants were significantly associated with at least one trait in one line. Figure 7a and b summarise the enrichment scores for all significant variants. The predicted consequence types that reached the greatest enrichment scores were LOF, frameshift indels, and unclassified missense variants, with various prevalence levels. Variants with intermediate prevalence levels were among the most enriched. These trends were accentuated when only considering candidate variants from haplotype blocks. In each line, we defined from 1554 to 2118 haplotype blocks. In total, across all lines and traits, 6692 candidate variants remained after accounting for linkage disequilibrium within each haplotype block. Figure 7c and d summarise the enrichment scores for the candidate variants. Enrichment scores based on the candidate variants revealed a stronger depletion of intergenic and intronic variants, and a much stronger enrichment for LOF, frameshift indels, and missense variants. For putatively functional variants, there were no clear trends of enrichment scores across prevalence levels. The trends of the enrichment scores between predicted consequence types and prevalence levels were similar for the three evaluated traits.

**Fig. 8:**
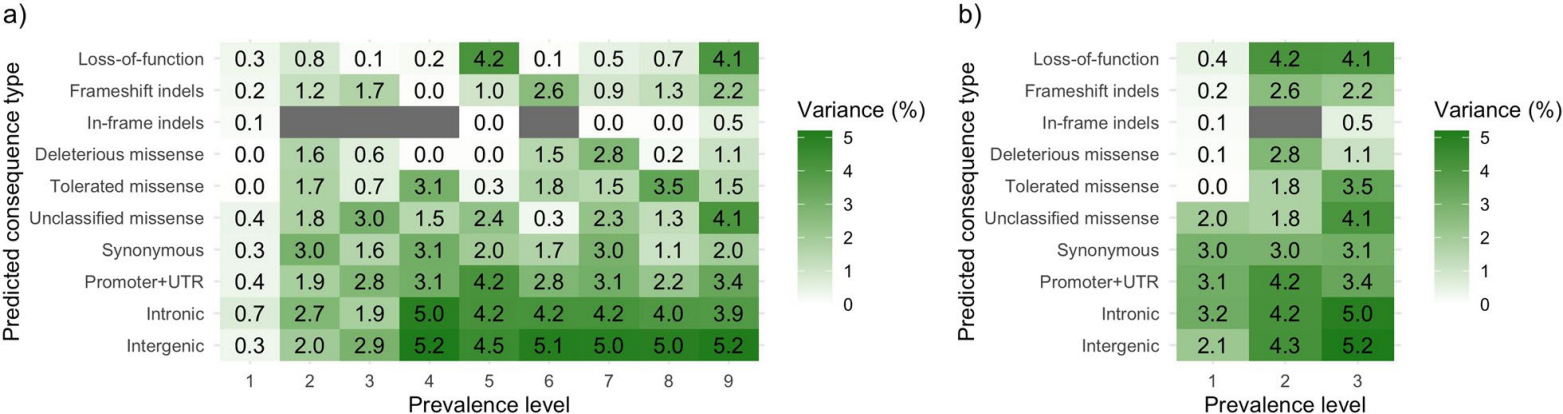
Maximum percentage of phenotypic variance explained by individual candidate variants within each prevalence level and predicted consequence type. Only the candidate variants after accounting for linkage disequilibrium were used. Prevalence level was considered across all nine lines (**a**) or only across the three lines included in the genome-wide association study (**b**)

**Fig. 9:**
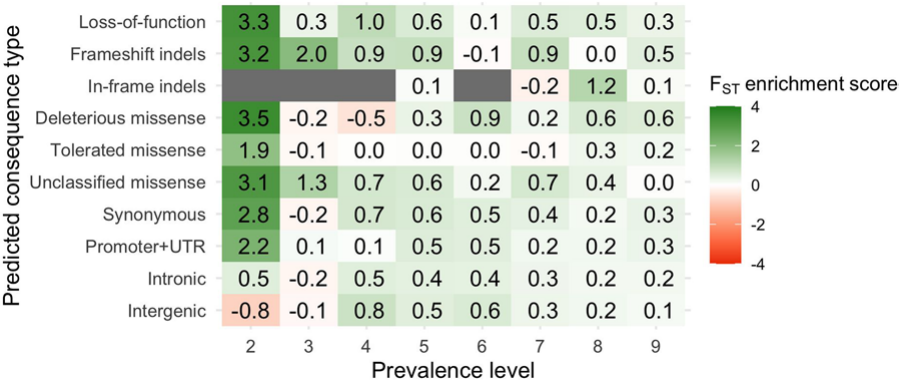
Enrichment score for the F_ST_ median of the candidate variants within each prevalence level and predicted consequence type. Only the candidate variants after accounting for linkage disequilibrium were used. Prevalence level was considered across all nine lines

In general, the lower allele frequency of low-prevalence variants hindered the detection of significant associations for these variants. Low-prevalence variants that were significantly associated with the evaluated traits actually had intermediate allele frequencies that were greater than expected for their prevalence level. Low-prevalence variants in general explained low percentages of variance (Fig. 8), although some low-prevalence variants explained up to 3.2% of phenotypic variance. Significant variants had higher F_ST_ than other variants of the same predicted consequence type and prevalence level (Fig. 9). The enrichment of significant variants for higher F_ST_ was especially strong for low-prevalence variants, which in some instances reached F_ST_ of ~ 0.15.

## Discussion

Our results contextualize the importance of population-specific and low-prevalence genetic variants. In the following, we will discuss: (1) the distribution and functional annotation of low-prevalence variants, (2) the load of putatively functional alleles by prevalence level, and (3) the association of low-prevalence variants with production traits.

### Distribution and functional annotation of low-prevalence variants

The main difficulty for the study of low-prevalence genetic variants is that the prevalence of a variant across lines is strongly related to its allele frequency in the line, such that the low-prevalence variants are also rare within the lines in which they occur. This is possibly because low-prevalence variants are relatively recent or constrained by negative selection.

On the one hand, the density of private variants was less correlated with recombination rate than the density of widespread variants and, therefore, regions with a low recombination rate were enriched for private variants. Although the interplay between recurring sweeps, background selection, and other phenomena at play is not fully understood, it is generally accepted that selection on linked variants leads to loss of variation in regions with low recombination rates [60]. Our observation that regions with a low recombination rate were enriched for private variants suggests that private variants may have been less affected by selective sweeps than widespread variants. This is consistent with previous observations of the younger age of rare and low-prevalence variants [61] and suggests that private variants tend to have arisen more recently than widespread variants, likely after line differentiation, and accumulated in low-recombining regions due to the reduced efficacy of purifying selection in those regions [62, 63].

On the other hand, low-prevalence variants were enriched for putatively functional variants with signs of a greater severity (stop-gain and frameshift indels that occur earlier in the transcript, and missense variants that were predicted to be deleterious). Variants that affect performance traits or that cause a detrimental condition are under directional selection and are therefore driven towards loss or fixation [64, 65]. The low F_ST_ estimates for the low-prevalence variants indicated that selection pressure keeps these variants at low minor allele frequencies even when they occur in several lines, especially if they are putatively functional [66]. This could be caused by natural selection or by similar selection objectives across livestock populations. These observations are also consistent with previous reports that some putatively functional variant categories (such as stop-gain and deleterious missense) are enriched for variants that are private to single cattle breeds [33], that putatively functional variants are less likely to have a high frequency of the alternative allele across multiple chicken lines [35], and that population-specific variants in non-African humans are enriched for putatively functional variants [67].

The relationship of variant prevalence across lines with allele frequency highlights the suitability of using a low-coverage sequencing approach to study this fraction of genetic variation. Nonetheless, bioinformatics pipelines for calling, genotyping, and even imputing such variants should account for the increased uncertainty because of their low allele frequency. We decided to use a very relaxed variant calling strategy with little filtering in order to account for as many rare variants as possible, but a sizeable fraction of these rare variants were discarded after imputation because they were fixed for the imputed individuals that passed quality control. Low-coverage sequencing is also not suitable for other types of genetic variants, such as structural variations (copy number variants, tandem duplications, and inversions), which could also be putatively functional and population-specific [68]. Of course, the number of called variants and the proportion that were private or widespread depend on the number of sequenced lines [32, 35] as well as on the sequencing effort in each line.

Our results also suggest that what is typically grouped as LOF is actually a heterogeneous category. In particular, frameshift indels showed patterns that did not conform to the other predicted consequence types.

### Load of putatively functional alleles by prevalence level

We found that an average individual carried a larger number of LOF and missense deleterious variants than previously reported in other livestock species or in humans. However, to date there is no clear consensus on the number of LOF and deleterious missense alleles that are present in the genome of an average individual. In humans, it has been estimated that an average individual carries 100 to 150 LOF alleles [64, 69–71] and around 800 weakly deleterious mutations [72], most of which are rare. The average number of LOF and deleterious alleles carried by an individual has been reported to be larger in domestic livestock populations than in wild populations [73], including estimates of 100 to 300 deleterious variants in domestic pigs [74], over 400 deleterious variants in domestic chicken [74], and 1200 to 1500 deleterious variants in domestic yak [75]. Similar magnitudes have been reported in dogs [76], rice [77], and sunflower [63].

It has been debated why healthy individuals carry a larger number of LOF variants in the homozygous state than expected [78, 79]. One possible reason is that not all predicted LOF variants are detrimental and their functional impact should be validated before being considered as such. Many predicted LOF variants are in fact neutral, advantageous (either in the wild or in controlled production environments), or may be the result of sequencing and annotation errors [78]. The claim that not all predicted LOF variants are detrimental is supported by the large proportion of LOF observed in the homozygous state for the alternative allele compared to the other consequence types, which casts doubt on the real impact of those variants. In contrast, individuals carried a lower proportion of alleles predicted to be deleterious missense in the homozygous state, which supports that such variants may have a real impact on genetic variation of production traits and, therefore, be subject to selection pressure.

These observations have implications for the identification of variants to be used for genomic prediction or for genomic edition strategies, such as promotion of alleles by genome editing (PAGE) [26] or removal of alleles by genome editing (RAGE) [27]. Efforts to promote or remove alleles should target variants that make a substantial contribution to traits of interest, i.e. functional variants. However, it is hard to computationally predict and statistically estimate the effects of such variants, especially if they have a low allele frequency. The number of LOF variants in the homozygous state for the alternative allele suggests that predicted loss of function is not a good indicator that a variant is strongly deleterious in the context of livestock breeding. Similarly, bioinformatic predictors of missense variant effects appear to be not very accurate [80, 81]. High-throughput fine-mapping and variant screening would be needed to ascertain variant causality and disentangle causality from linkage disequilibrium.

### Associations of low-prevalence variants with production traits

Genome-wide association studies for three polygenic traits of economic importance in the three largest lines revealed that the variants with significant associations were enriched for putatively functional roles, such as LOF, frameshift indels, and missense variants, and depleted of intergenic variants. This pattern of enrichment was similar to previous reports from human datasets [82]. However, only a few of the population-specific and low-prevalence variants were significantly associated with the traits, even after accounting for linkage disequilibrium. Most of the significant variants showed intermediate or high prevalence levels, which is consistent with previous meta-analyses in cattle that showed that significant variants are often common variants [83]. This could be because quantitative trait nucleotides have intermediate or high allele frequencies or because most studies are underpowered to map rare causal variants. The latter may be more likely given that the significant private and low-prevalence variants had intermediate allele frequencies. Although it cannot be ruled out that the significant low-prevalence variants reached intermediate allele frequencies by drift or by hitchhiking with linked variants under selection [84], it is plausible that these variants have biological functions that contribute to trait phenotypic variance. However, these variants amounted to a small number of variants that generally explained small fractions of variance.

Determining which of the variants that are in linkage disequilibrium is the most likely to be causal remains one of the greatest challenges in genomics. Here we prioritised the most severe variants within each haplotype block, which were more likely to have a low prevalence, as candidate variants. However, other more widespread variants, including intergenic variants, that were in high linkage disequilibrium with the significant low-prevalence variants successfully acted as tag variants and captured much larger fractions of trait variance. This makes the widespread variants more suitable for applications in animal breeding and justifies their inclusion in tools such as marker arrays. A similar result was found in cattle, where splice site and synonymous variants explained the largest proportions of trait variance, while missense variants explained almost no variance [85]. It is worth pointing out that even a variant with a large allele substitution effect will explain a small percentage of variance if the minor allele is rare.

It is conceivable that some of the low-prevalence variants with a low allele frequency have non-negligible effects for traits of interest. In spite of the large number of individuals included in this study, the large number of variants investigated and the pervasiveness of linkage disequilibrium among them still make disentangling their contribution to trait variance very challenging. While genome-wide association studies that involve more than one breed typically find multiple breed-specific associations (e.g., [86]), based on our results it seems unlikely that breed-specific associations arise from the low-prevalence variants. Instead, breed-specific associations depend on the effect of the differences in allele frequencies, linkage disequilibrium structure, and other genetic background features on the power to detect the effect of prevalent variants across populations. Significant variants had higher F_ST_ estimates than non-significant variants, which is also consistent with previous reports [83]. Although the enrichment for higher F_ST_ was greater for low-prevalence variants, it remains unclear to which degree the significant low-prevalence variant with high F_ST_ explain differences among lines for the studied traits or their allele frequency reflect selection history.

## Conclusions

Low-prevalence variants are enriched for putatively functional variants, including LOF and deleterious missense variants. However, most low-prevalence variants are kept at very low allele frequencies by negative selection or because they have arisen more recently than other higher-prevalence variants. Only a small subset of low-prevalence variants had intermediate allele frequencies and large estimated effects on production traits. Low-prevalence variants that were significantly associated with complex traits had greater degrees of differentiation between lines (per-site F_ST_) than non-significant variants in the same category. However, more widespread variants, including intergenic variants, captured larger proportions of trait variance. Therefore, overall, accounting for population-specific and other low-prevalence variants is unlikely to noticeably benefit across-breed analyses, such as the prediction of genomic breeding values in a population using reference populations of a different genetic background.

## Supplementary Information

**Additional file 1: Figure S1.** Population structure of the sequenced pigs according to the two first principal components. The colour clusters correspond to lines A to I.

**Additional file 2: Supplementary Methods.** Complete description of the quality control criteria that were applied on the total number of variants called.

**Additional file 3: Table S1.** Number of analysed variants by chromosome.

**Additional file 4: Figure S2.** Variant density for the private variants in each line.

## Data Availability

The software packages AlphaPhase, AlphaImpute, and AlphaPeel are available from https://github.com/AlphaGenes. The software package AlphaSeqOpt is available from the AlphaGenes website (http://www.alphagenes.roslin.ed.ac.uk). The datasets generated and analysed in this study are derived from the PIC breeding programme and not publicly available.
