# Supplementary material for: Rare and population-specific functional variation across pig lines
Source: Genet Sel Evol. 2022 Jun 3;54:39. doi: 10.1186/s12711-022-00732-8 (PMC9164375; doi:10.1186/s12711-022-00732-8)
Supplement: Supplementary file 1 — Additional file 1: Figure S1. Population structure of the sequenced pigs according to the two first principal components. The colour clusters correspond to lines A to I. [file 12711_2022_732_MOESM1_ESM.pdf]

Additional File 1

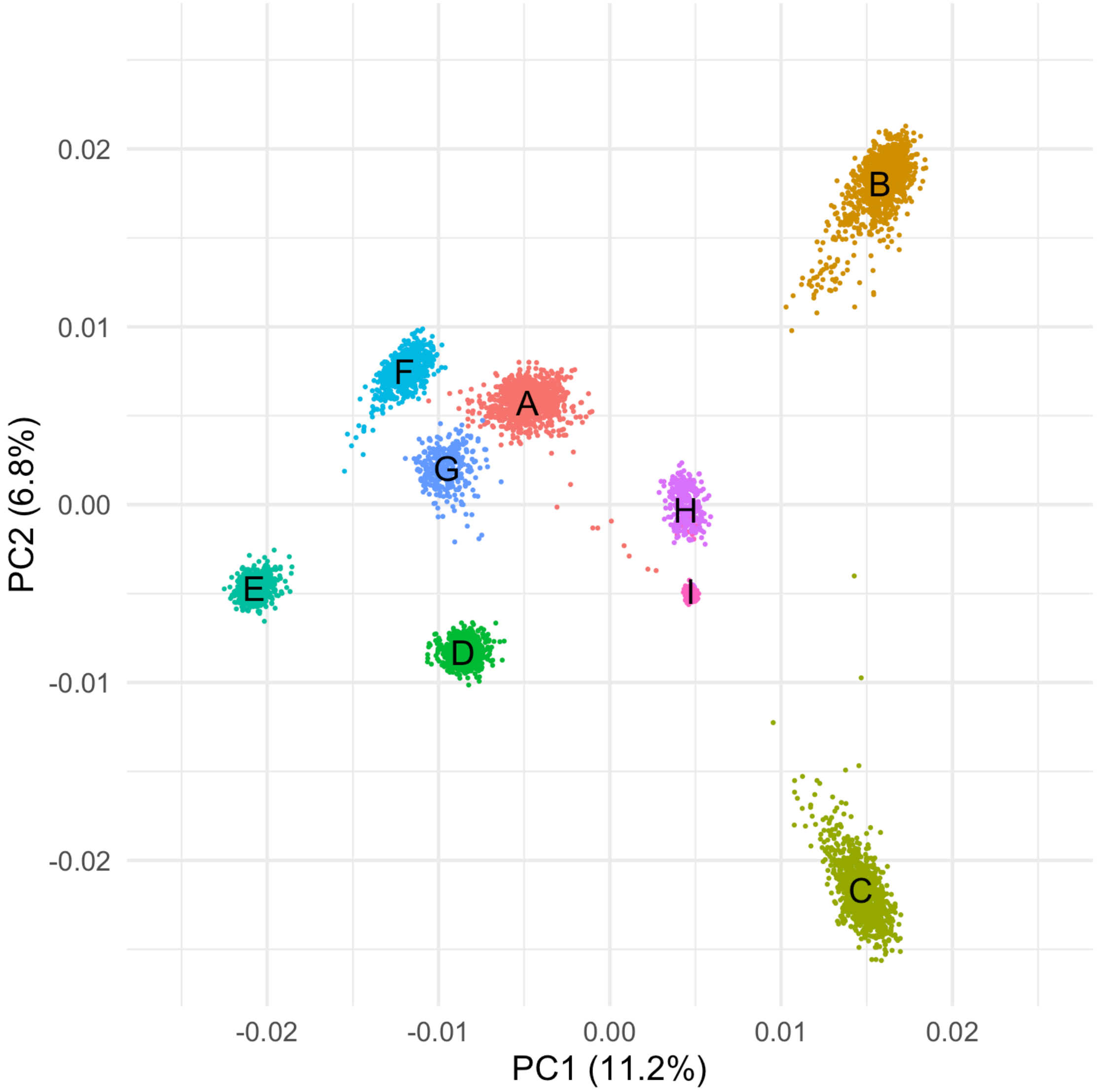

Figure S1. Population structure of the sequenced pigs according to the two first principal components. The colour clusters correspond to lines A to I.
