# Supplementary material for: Rare and population-specific functional variation across pig lines
Source: Genet Sel Evol. 2022 Jun 3;54:39. doi: 10.1186/s12711-022-00732-8 (PMC9164375; doi:10.1186/s12711-022-00732-8)
Supplement: Supplementary file 2 — Additional file 2: Supplementary Methods. Complete description of the quality control criteria that were applied on the total number of variants called. [file 12711_2022_732_MOESM2_ESM.pdf]

Additional File 2:

Supplementary Methods

A total of 70,739,387 variants were called across all nine lines. Of these, 24,394,763 variants failed to meet quality control criteria. Of these, 148,825 variants were discarded because they had mean depth values 3 times greater than the average realized coverage, 1,927,221 were multiallelic within line, and 1,673,219 were biallelic within line but multiallelic when all lines were considered. The remaining variants were imputed for all pedigreed individuals, but 20,645,588 of them were fixed for the reference allele in the imputed individuals that passed our accuracy quality control. This affected mostly variants that had been called in only one line and for which the alternative allele segregated at very low frequency. The hypothesis that such variants arise from false positives in variant calling seems unlikely to be the main cause as for more than 99% of these variants we read the alternative allele in at least two individuals. Additionally, we previously quantified that 96.9% of the variants called from low-coverage data were confirmed by sequencing the same individuals at high coverage [1]. A total of 46,344,624 biallelic variants passed quality control criteria across all lines.

1. Ros-Freixedes R, Battagin M, Johnsson M, Gorjanc G, Mileham AJ, Rounsley SD, & Hickey JM. 2018. Impact of index hopping and bias towards the reference allele on accuracy of genotype calls from low-coverage sequencing. Genet Sel Evol, 50: 64.
