# Supplementary material for: Rare and population-specific functional variation across pig lines
Source: Genet Sel Evol. 2022 Jun 3;54:39. doi: 10.1186/s12711-022-00732-8 (PMC9164375; doi:10.1186/s12711-022-00732-8)
Supplement: Supplementary file 3 — Additional file 3: Table S1. Number of analysed variants by chromosome. [file 12711_2022_732_MOESM3_ESM.pdf]

Additional File 3

Table S1. Number of analysed variants by chromosome.

Chromosome	Length (Mb)	SNPs (M)	Indels (M)	Variant density (thousands/Mb)
1	274.3	3.77	0.76	16.5
2	151.9	2.60	0.52	20.5
3	132.8	2.35	0.44	21.0
4	130.9	2.21	0.43	20.2
5	104.5	1.95	0.39	22.4
6	170.8	2.80	0.55	19.6
7	121.8	2.20	0.43	21.6
8	139.0	2.37	0.50	20.6
9	139.5	2.47	0.48	21.1
10	69.4	1.60	0.31	27.5
11	79.2	1.57	0.31	23.7
12	61.6	1.35	0.25	26.0
13	208.3	2.97	0.64	17.3
14	141.8	2.38	0.48	20.2
15	140.4	2.20	0.46	18.9
16	79.9	1.50	0.30	22.5
17	63.5	1.32	0.25	24.7
18	56.0	1.04	0.19	22.0
Total	2,501.9	38.64	7.70	18.5
