# Supplementary figures and images for: Rare and population-specific functional variation across pig lines
Source: Genet Sel Evol. 2022 Jun 3;54:39. doi: 10.1186/s12711-022-00732-8 (PMC9164375; doi:10.1186/s12711-022-00732-8)

**Additional File 4**

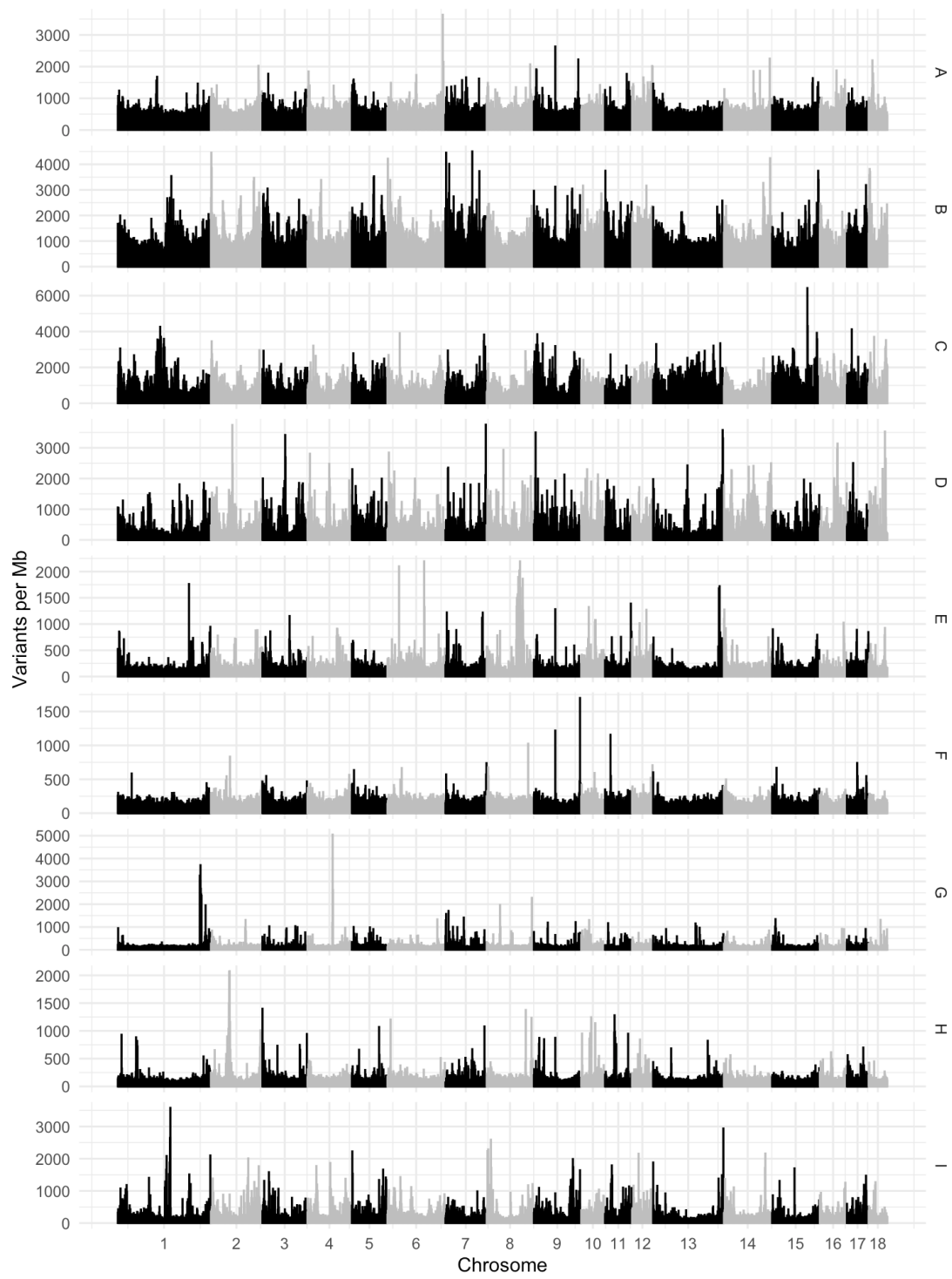

**Figure S2.** Variant density for the private variants in each line.

Supplement: Supplementary file 4 — Additional file 4: Figure S2. Variant density for the private variants in each line. [file 12711_2022_732_MOESM4_ESM.pdf]
